# Supplementary material for: Identification and functional analysis of non-coding regulatory small RNA FenSr3 in Bacillus amyloliquefaciens LPB-18
Source: PeerJ. 2023 May 15;11:e15236. doi: 10.7717/peerj.15236 (PMC10194069; doi:10.7717/peerj.15236)
Supplement: Supplemental Information 4 [file peerj-11-15236-s004.zip › KO/CK-vs-T1_map/map00910.html]

KEGG PATHWAY: Nitrogen metabolism - Reference pathway


|  |  |
| --- | --- |
| **Nitrogen metabolism - Reference pathway** |  |

[
Pathway menu
| Organism menu
| Pathway entry
| Show description
| User data mapping
]

The biological process of the nitrogen cycle is a complex interplay among many microorganisms catalyzing different reactions, where nitrogen is found in various oxidation states ranging from +5 in nitrate to -3 in ammonia. The core nitrogen cycle involves four reduction pathways and two oxidation pathways. Nitrogen fixation [MD:M00175] is the process of reducing atmospheric molecular nitrogen to ammonia, a biologically useful reduced form incorporated into amino acids and other vital compounds. The ability of fixing atmospheric nitrogen by the nitrogenase enzyme complex is present in restricted prokaryotes (diazotrophs). The other reduction pathways are assimilatory nitrate reduction [MD:M00531] and dissimilatory nitrate reduction [MD:M00530] both for conversion to ammonia, and denitrification [MD:M00529]. Denitrification is a respiration in which nitrate or nitrite is reduced as a terminal electron acceptor under low oxygen or anoxic conditions, producing gaseous nitrogen compounds (N2, NO and N2O) to the atmosphere. The two oxidation pathways are nitrification [MD:M00528] and anaerobic ammonium oxidation (anammox). Nitrification is the oxidation of ammonia (NH3) with oxygen into nitrite followed by the oxidation of nitrite into nitrate. The first step is performed by ammonia-oxidizing microorganisms (e.g., Nitrosomonas and Nitrosococcus) and the second step by nitrite-oxidizing microorganisms (e.g., Nitrobacter). Anammox is a biochemical process of oxidizing ammonium (NH4+) into dinitrogen gas (N2) using nitrite as an electron acceptor. It occurs in the anammoxosome, a membrane bound compartment inside the cytoplasm, of anammox bacteria (e.g., Planctomycetes).

|  |  |  |
| --- | --- | --- |
| Reference pathway | 184% 150% 122% 100% 82% 67% 55% | 图片下载 |
